# Prolonged Mechanical Ventilation in Patients with Deep-Seated Intracerebral Hemorrhage: Risk Factors and Clinical Implications

**DOI:** 10.3390/jcm10051015

**Published:** 2021-03-02

**Authors:** Felix Lehmann, Lorena M. Schenk, Inja Ilic, Christian Putensen, Alexis Hadjiathanasiou, Valeri Borger, Julian Zimmermann, Erdem Güresir, Hartmut Vatter, Christian Bode, Matthias Schneider, Patrick Schuss

**Affiliations:** 1 Department of Anesthesiology and Intensive Care Medicine, University Hospital Bonn, 53127 Bonn, Germany; Christian.putensen@ukbonn.de (C.P.); christian.bode@ukbonn.de (C.B.); 2 Department of Neurosurgery, University Hospital Bonn, 53127 Bonn, Germany; lorena_maria.schenk@ukbonn.de (L.M.S.); inja.ilic@ukbonn.de (I.I.); alexis.hadjiathansiou@ukbonn.de (A.H.); valeri.borger@ukbonn.de (V.B.); erdem.gueresir@ukbonn.de (E.G.); hartmut.vatter@ukbonn.de (H.V.); matthias.schneider@ukbonn.de (M.S.); patrick.schuss@ukbonn.de (P.S.); 3 Department of Neurology, University Hospital Bonn, 53127 Bonn, Germany; julian.zimmermann@ukbonn.de

**Keywords:** prolonged mechanical ventilation, intracerebral hemorrhage, intensive care

## Abstract

While management of patients with deep-seated intracerebral hemorrhage (ICH) is well established, there are scarce data on patients with ICH who require prolonged mechanical ventilation (PMV) during the course of their acute disease. Therefore, we aimed to determine the influence of PMV on mortality in patients with ICH and to identify associated risk factors. From 2014 to May 2020, all patients with deep-seated ICH who were admitted to intensive care for >3 days were included in further analyses. PMV is defined as receiving mechanical ventilation for more than 7 days. A total of 42 out of 94 patients (45%) with deep-seated ICH suffered from PMV during the course of treatment. The mortality rate after 90 days was significantly higher in patients with PMV than in those without (64% versus 22%, *p* < 0.0001). Multivariate analysis identified “ICH volume >30 mL” (*p* = 0.001, OR 5.3) and “admission SOFA score > 5” (*p* = 0.007, OR 4.2) as significant and independent predictors for PMV over the course of treatment in deep-seated ICH. With regard to the identified risk factors for PMV occurrence, these findings might enable improved guidance of adequate treatment at the earliest possible stage and lead to a better estimation of prognosis in the course of ICH treatment.

## 1. Introduction

Spontaneous intracerebral hemorrhage (ICH) is a major contributor to mortality and morbidity in stroke patients [1,2,3]. Despite ongoing efforts to improve surgical and medical management, advances in mortality and morbidity of this disease are modest [1,4,5]. Even with the best medical management and/or surgical treatment, functional outcomes remain poor, with less than 20% of all ICH patients regaining functional independence after 6 months [6]. Depending on the localization, ICH is differentiated into deep-seated ICH—involving the basal ganglia and/or thalamus—and superficial, lobar ICH. The different localizations of ICH may have various causes [6]. Thus, deep-seated ICH is often associated with hypertensive long-term damage, while lobar ICH may have other bleeding sources (aneurysm, arteriovenous malformation (AVM), cerebral amyloid angiopathy (CAA), tumor, infection) [2]. The resulting heterogeneity in these cases makes ICH generally difficult to study.

Along with the bleeding related damage that often occurs to eloquent brain tissue, ICH might also lead to a long-lasting impairment of vigilance, which often results in subsequent prolonged dependence on a ventilator [7]. The condition known as prolonged mechanical ventilation (PMV) has already been identified as an important factor influencing mortality in numerous other etiologies/specialties [8,9,10,11]. Nevertheless, the influence of PMV in patients with ICH and the possible risk factors for PMV in these patients remains to be clarified.

Therefore, in the present study we investigated a cohort of patients with deep-seated ICH only—also in terms of minimized heterogeneity—for risk factors for the necessity of PMV and its potential impact on mortality.

## 2. Materials and Methods

Clinical records of patients who were admitted to the neurosurgical department of the University Hospital Bonn between January 2014 and May 2020 for non-traumatic intracerebral spontaneous hemorrhages were retrospectively evaluated. After study approval by the local institutional review board (IRB), patients with lobar ICH and/or ICH with associated cause of bleeding (e.g., aneurysm, arteriovenous malformation, trauma) were excluded from further evaluation. Only patients with deep-seated ICH were considered for further analysis. ICH localization was deemed deep-seated if the hemorrhage originated predominantly in or involved the basal ganglia or thalamus [12,13,14]. Information collected for each patient included patient characteristics, pre-existing conditions, ICH location, ICH volume [15], neurological status at admission, ICH score [3], Intensive Care Unit (ICU) and laboratory admission parameters, admission SOFA/SAPS scores [16,17], necessity of prolonged mechanical ventilation (PMV), 30-day mortality, and 3-month outcomes/mortality and treatment strategies chosen during hospitalization.

The best medical treatment was provided to all patients suffering from deep-seated ICH according to the hospitals internal standard operating procedures, which are in accordance with the guidelines of the American Heart Association/American Stroke Association [1]. Best medical treatment included, among other procedures, rapid resolution of severe coagulation factor deficiencies or severe thrombocytopenia, normalization of blood pressure, implementation of general monitoring, and management of any elevated intracranial pressure.

The first spontaneous breathing trial was performed within at least 5 h after arrival at the ICU. Patients were evaluated as eligible for extubation if they presented adequate oxygenation and ventilation indices, sustained hemodynamic stability and demonstrated minimum neurological function including logopedic evaluation of dysphagia. The final decision to extubate, re-intubate or perform percutaneous tracheostomy was based on the independent discretion of the treating intensive care physician. PMV was defined as the inability to wean from the ventilator more than 7 days after admission. The cut-off at 7 days was chosen in accordance with the definition proposed by the Task Force of the European Respiratory Society and respective previous studies [8,18].

Dividing values that could be used to dichotomize patients with respect to the different variables had already been defined previously: ICH volume [3], Glasgow Coma Scale (GCS) [3], ICH score [3], c-reactive protein (CRP) [19], white blood cells (WBC) [19]. The optimal cut-off values for the SOFA and SAPS values were defined separately for the investigated patient population, as described in the following.

The modified Rankin Scale (mRS) was used to assess the functional outcome. Patients were dichotomized after mRS in two groups: (1) favorable outcome (mRS 0–4) versus (vs.) unfavorable outcome (mRS 5–6), as previously defined [20].

Data analysis was performed using the computer software package SPSS (version 25, IBM Corp., Armonk, NY, USA). The Mann–Whitney U-test was chosen to compare continuous variables as the data were mostly not normally distributed. An unpaired *t*-test was used for parametric statistics after testing for normal distribution. Categorical variables were analyzed in contingency tables using Fisher’s exact test. Results with *p* < 0.05 were considered statistically significant. Regarding the optimal cut-off values of the SOFA and SAPS scores to differentiate between patients with the need for PMV and those without PMV, an appropriate approximation was attempted after determining the area under the curve (AUC) using constructed receiver operating characteristic (ROC) curves with the aid of Youden’s index.

In addition, in order to determine independent predictors of the necessity of PMV in patients with deep-seated ICH, a multivariate analysis using binary logistic regression was performed. Therefore, a backward stepwise method was used to construct a multivariate logistic regression model in relation to PMV as a dependent variable with an inclusion criterion for variables with presumed/proven clinical relevance. The variables included in the multivariate analysis were the following: ICH volume, SOFA, SAPS, acute kidney injury (AKI).

## 3. Results

### 3.1. Patient Characteristics

A total of 94 patients with deep-seated spontaneous ICH were identified and further analyzed, as described below. The median volume of hemorrhage in the studied patients with deep-seated ICH was 31.6 mL (IQR 14.4–68.9). Surgical management of patients with deep-seated ICH in the reported patient cohort included stereotactic aspiration of the hemorrhage in seven patients (7%), decompressive hemicraniectomy (DC) with ICH evacuation in three patients (3%), and DC without ICH evacuation in 19 patients (20%). Cerebrospinal fluid diversion as a sole surgical intervention was performed in 26 patients (28%). Overall, 42 of 94 patients (45%) required prolonged mechanical ventilation > 7 days after admission. With regard to patient age, there was no significant difference observed between patients with and without PMV (Table 1). Additionally, with regard to pre-existing comorbidities and/or the administration of anticoagulant medication prior to bleeding, no significant difference could be identified between the groups with and without PMV. Further details are given in Table 1.

### 3.2. Admission Characteristics in Deep-Seated ICH Patients

Patients with subsequent PMV after deep-seated ICU presented with significantly larger intracerebral hematomas compared to patients without PMV (*p* < 0.0001) (Table 2). Furthermore, patients with the necessity of PMV presented with an initial Glasgow Coma Scale (GCS) > 12 significantly less often compared to patients without PMV (*p* = 0.0002, OR 5.8, 95% CI 2.2–15.5). Neither the presence of intraventricular blood nor the results of the initial assessment of the ICH score differed significantly between the two groups of patients with and without PMV (Table 2).

### 3.3. ICU Admission Parameters

Patients with subsequent PMV after deep-seated ICU suffered significantly more often from early acute kidney injury (AKI; within 48 h after admission) compared with patients without PMV (*p* = 0.02, OR 3.6, 95% CI 1.3–9.9) (Table 2). With a sensitivity of 91% and a specificity of 52%, a cut-off point at 30 was identified for the admission SAPS score (AUC 0.78, *p* < 0.0001). Subsequently, patients with PMV presented significantly more often with a SAPS score >30 compared to patients without PMV during the treatment course (*p* < 0.0001, OR 5.4, 95% CI 2.0–14.3). With a sensitivity of 43% and a specificity of 92%, a cut-off point at five was identified for the admission SOFA score (AUC 0.79, *p* < 0.0001). Subsequently, patients with PMV presented significantly more often with a SOFA score > five compared to patients without PMV during the treatment course (*p* < 0.0001, OR 16.0, 95% CI 4.9–52.5; Table 2).

### 3.4. Impact of PMV on Length of Stay/Outcome/Mortality

Patients with PMV after deep-seated ICH had significantly longer hospital stays compared to patients without PMV (34 ± 21 days versus 17 ± 37 days, *p* < 0.0001). Patients with PMV developed ventilator-associated pulmonary complications significantly more often compared to patients without PMV (45% versus 14%; *p* = 0.001, OR 5.3, 95% CI 1.9–14.5). Patients with PMV achieved a favorable outcome (mRS 0–4) after 3 months significantly less often when compared with patients without PMV and deep-seated ICH (36% vs. 73%; *p* < 0.0001, OR 4.9, 95% CI 2.0–11.8) (Table 2). Using a more stringent outcome comparison, patients with PMV also experienced a worse outcome (mRS 3–6) compared to patients without PMV (*p* = 0.04). Additionally, the 90-days-mortality rate in patients with PMV and deep-seated ICH was significantly higher compared to patients without PMV and deep-seated ICH (64% vs. 25%; *p* < 0.0001, OR 5.4, 95% CI 2.2–13.2; Table 2).

### 3.5. Multivariate Analysis

**Figure 1:**
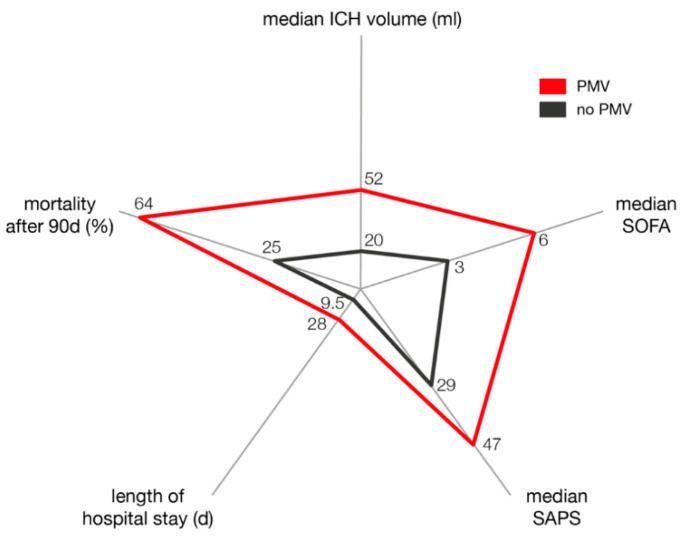
Graphical display of selected ICU admission data in PMV/non-PMV patients and corresponding impact on length of hospital stay and mortality after 90 days.

The multivariate analysis identified “ICH volume > 30 mL” (*p* = 0.001, OR 5.7, 95% CI 1.9–16.7) and “admission SOFA score > 5” (*p* < 0.0001, OR 15.2, 95% CI 4.3–54.6) as independent predictors for the necessity of PMV in patients suffering from deep-seated ICH (Nagelkerke’s R2 = 0.47; Figure 1).

## 4. Discussion

The profound impact of intracerebral hemorrhage on both neurological outcome and mortality has been well studied [21,22]. Especially for the initial assessment of patients with ICH, numerous factors as well as scores have been established [3,23,24]. The often irreversible neurological damage caused by the bleeding, however, often confronts the affected patients themselves or their relatives/caregivers with the difficult decision to initiate/conduct the therapeutic process with intensive care measures or to opt for withdrawal of life-sustaining therapy [4,25,26,27]. Ideally, patients known wishes, personal preferences, value systems, and advance directives—if available—should be considered in such decision-making processes. In everyday clinical practice, however, such decisions are largely dependent on the prognosis assessment of the treating physician team and the derived goals-of-care [28]. Thus, a detailed analysis of possible predictors for unfavorable outcome after ICH is necessary for this prognosis estimation. In addition to the above-mentioned clinical scores and individual factors, prediction models are increasingly being established to provide a basis for decision-making in this process [28]. Therefore, in the present study we were able to illustrate a significant influence of prolonged mechanical ventilation on clinical outcome and/or mortality in our selected cohort of patients with deep-seated ICH.

PMV has been proven to have a negative impact on outcome as well as survival, and is associated with incremental health care costs across various specialties and etiologies [9,11,29,30,31]. In addition to the extensive implications that prolonged mechanical ventilation might have on the outcome, these are often not due to the actual ventilation, but to its further-reaching complications. For example, in the present patient population, ventilator-associated pulmonary complications (e.g., pneumonia) occurred in 45% of patients who required PMV, but only in 14% of patients without PMV. This circumstance is associated with subsequent further therapy (here: antibiotic therapy), which in turn might lead to further complications. However, to the best of our knowledge, this is the first study that has focused on the predictors and influence of PMV in patients with ICH. In accordance with Saber et al. in the setting of PMV following endovascular stroke therapy, we consider PMV as an important independent clinical outcome, in contrast to previous studies, and therefore, focused on predictors of PMV based on initial clinical characteristics [10].

In the present study, the initial SOFA score is an important predictor for a subsequent need for PMV. The SOFA scoring system has proven to be a useful predictor of ICU mortality, although it was not designed to predict, but rather to describe, a sequence of complications in critically ill patients [17,32]. However, evaluation of a score that measures/monitors organ dysfunction should take into account that organ failure is not an all-or-nothing phenomenon, but rather a continuum of changes in organ function leading to alterations over time [33]. Nevertheless, the initial SOFA score at ICU admission already delivers a good estimate of later mortality of the critically-ill patient [33]. In the present study, an initial SOFA score > five has been identified as a significant and independent predictor of the need for PMV in patients with deep-seated ICH.

Another predictor for the necessity of PMV in the present study was the volume of the intracranial hematoma in patients suffering from deep-seated ICH. As a part of the established ICH score of Hemphill et al., hematoma size is an established measure of mortality in patients with ICH [3]. In addition to pulmonary causes, impairment of consciousness is most likely responsible for a prolonged weaning from mechanical ventilation [7]. The association between reduced consciousness and increased ICH volume has been described previously [7,34,35]. With regard to the necessity of ventilation and thus the timing of tracheotomy, the SETPOINT score of Bösel et al., for example, also considers hematoma size in patients with ICH [36]. In the present study, an initial ICH volume > 30 mL was identified as a significant and independent predictor of the need for PMV in patients with deep-seated ICH.

We aimed at identifying potential predictors of prolonged mechanical ventilation in patients with deep-seated intracerebral hemorrhage. Both ICH volume and SOFA score at ICU admission are of crucial importance in this context. Therefore, the results of the present study are intended to support physicians in formulating goals-of-care and/or patient-adapted treatment decisions.

The present study has several limitations. The data collection was conducted retrospectively and only includes a single center’s experience. Patients were not randomized, but treated according to the preferences of the treating physicians. In addition, the homogeneous, but therefore, small patient population makes it difficult to interpret the multivariate analysis performed, without introducing the risk of bias and increased variability. Nevertheless, the present study investigates an important aspect in a selected, and thus, less confounder-prone patient cohort and therefore provides the basis for the initiation of multicenter registries and/or further studies.

## 5. Conclusions

The present study in a highly selective patient cohort reveals that patients with deep-seated ICH have a high incidence of PMV. Increased ICH volume and an elevated SOFA score at ICU admission were identified as predictors. Knowledge of potential risk factors is essential to improve early initiation of adequate treatment and prognosis assessment in the early stages of ICH treatment.

## Figures and Tables

**Table 1:** Characteristics for patients with deep-seated intracerebral hemorrhage (ICH).

	Patients without PMV (*n* = 52)	Patients with PMV (*n* = 42)	
Mean age (years)	65 ± 16	64 ± 11	n.s.
female sex	21 (%)	12 (%)	n.s.
pre-existing hypertension	41 (%)	37 (%)	n.s.
diabetes mellitus	5 (10%)	7 (17%)	n.s.
previous stroke	9 (%)	8 (%)	n.s.
chronic obstructive pulmonary disease COPD	3 (6%)	5 (12%)	n.s.
anticoagulant medication prior ictus	23 (44%)	17 (41%)	n.s.

**Table 2:** ICU admission parameters and outcome.

	Patients without PMV (*n* = 52)	Patients with PMV (*n* = 42)	Significance
median ICH volume (IQR, mL)	20.3 (9.9–39.6)	51.8 (29.7–93.9)	*p* < 0.0001
ICH volume > 30 mL	18 (35%)	32 (76%)	*p* < 0.0001, OR 6.0, 95% CI 2.4–15.0
admission GCS > 12	28 (54%)	7 (17%)	*p* = 0.0002, OR 5.8, 95% CI 2.2–15.5
ICH score > 3	6 (12%)	11 (26%)	n.s.
admission CRP > 10 mg/L	13 (25%)	11 (26%)	n.s.
admission PCT > 0.5 μg/L	2 (4%)	7 (17%)	n.s.
admission WBC > 12 G/L	16 (31%)	19 (45%)	n.s.
early AKI	7 (14%)	15 (36%)	*p* = 0.02, OR 3.6, 95% CI 1.3–9.9
admission SAPS score > 30	25 (48%)	35 (83%)	*p* < 0.0001, OR 5.4, 95% CI 2.0–14.3
admission SOFA score > 5	4 (8%)	24 (57%)	*p* < 0.0001, OR 16, 95% CI 4.9–52.5
favorable outcome after 3 months (mRS 0–4)	38 (73%)	15 (36%)	*p* < 0.0001, OR 4.9, 95% CI 2.0–11.8
90 d mortality	13 (25%)	27 (64%)	*p* < 0.0001, OR 5.4, 95% CI 2.2–13.2

## Data Availability

Data are contained within the article.
